# Linkage and retention in HCV care for HIV‐infected populations: early data from the DAA era

**DOI:** 10.1002/jia2.25051

**Published:** 2018-04-10

**Authors:** Rachel Sacks‐Davis, Joseph S Doyle, Andri Rauch, Charles Beguelin, Alisa E Pedrana, Gail V Matthews, Maria Prins, Marc van der Valk, Marina B Klein, Sahar Saeed, Karine Lacombe, Nikoloz Chkhartishvili, Frederick L Altice, Margaret E Hellard

**Affiliations:** ^1^ Disease Elimination Program Burnet Institute Melbourne Australia; ^2^ Department of Medicine The University of Melbourne Melbourne Australia; ^3^ Department of Infectious Diseases The Alfred and Monash University Melbourne Australia; ^4^ Department of Infectious Diseases University Hospital and University of Bern Bern Switzerland; ^5^ Department of Epidemiology and Preventive Medicine Monash University Melbourne Australia; ^6^ The Kirby Institute University of New South Wales Sydney Australia; ^7^ Public Health Service Amsterdam Amsterdam the Netherlands; ^8^ International Antiviral Therapy Evaluation Center and Department of Infectious Diseases Tropical Medicine and AIDS Academic Medical Centre Amsterdam the Netherlands; ^9^ Division of Infectious Diseases and Chronic Viral Illness Service McGill University Health Centre Montreal Canada; ^10^ Department of Epidemiology, Biostatistics and Occupational Health McGill University Montreal Canada; ^11^ Infectious Diseases, AP‐HP Sorbonne Universités and Inserm UMR‐S1136 Paris France; ^12^ Infectious Diseases AIDS and Clinical Immunology Research Center Tbilisi Georgia; ^13^ Section of Infectious Diseases Yale School of Medicine New Haven CT USA; ^14^ Epidemiology of Microbial Diseases Yale School of Public Health New Haven CT USA; ^15^ Centre of Excellence in Research in AIDS University of Malaya Kuala Lumpur Malaysia

**Keywords:** hepatitis C virus, disease elimination, MSM, IDU, implementation science, barriers, cascade of care

## Abstract

**Introduction:**

There is currently no published data on the effectiveness of DAA treatment for elimination of HCV infection in HIV‐infected populations at a population level. However, a number of relevant studies and initiatives are emerging. This research aims to report cascade of care data for emerging HCV elimination initiatives and studies that are currently being evaluated in HIV/HCV co‐infected populations in the context of implementation science theory.

**Methods:**

HCV elimination initiatives and studies in HIV co‐infected populations that are currently underway were identified. Context, intervention characteristics and cascade of care data were synthesized in the context of implementation science frameworks.

**Results:**

Seven HCV elimination initiatives and studies were identified in HIV co‐infected populations, mainly operating in high‐income countries. Four were focused mainly on HCV elimination in HIV‐infected gay and bisexual men (GBM), and three included a combination of people who inject drugs (PWID), GBM and other HIV‐infected populations. None were evaluating treatment delivery in incarcerated populations. Overall, HCV RNA was detected in 4894 HIV‐infected participants (range within studies: 297 to 994): 48% of these initiated HCV treatment (range: 21% to 85%; within studies from a period where DAAs were broadly available the total is 57%, range: 36% to 74%). Among studies with treatment completion data, 96% of 1109 initiating treatment completed treatment (range: 94% to 99%). Among those who could be assessed for sustained virological response at 12 weeks (SVR12), 1631 of 1757 attained SVR12 (93%, range: 86% to 98%).

**Conclusions:**

Early results from emerging research on HCV elimination in HIV‐infected populations suggest that HCV treatment uptake is higher than reported levels prior to DAA treatment availability, but approximately half of patients remain untreated. These results are among diagnosed populations and additional effort is required to increase diagnosis rates. Among those who have initiated treatment, completion and SVR rates are promising. More data are required in order to evaluate the effectiveness of these elimination programmes in the long term, assess which intervention components are effective, and whether they need to be tailored to particular population groups.

## 1 Introduction

Over two million people are estimated to be HIV/HCV co‐infected globally 1. Chronic viral hepatitis accounts for approximately 10% of mortality among people living with HIV 2. Injecting drug use is the major risk factor for HCV acquisition among those living with HIV, accounting for over 60% of infections globally, whereas in many high‐income countries, high‐risk sexual behaviour among HIV‐infected gay and bi‐sexual men (GBM), including injecting and non‐injecting drug use to enhance the sexual experience, is a key driver of HCV transmission 1, 3, 4. Incarcerated populations are also at elevated risk of HCV and HIV infection due to incarceration of people who inject drugs (PWID) 5.

HCV treatment has been transformed through direct‐acting antiviral (DAA) medications that cure >90% of individuals using tablets over 8 to 12 weeks 6. Despite modelling studies suggesting that if HCV treatment can be adequately scaled to need, HCV prevalence and incidence can be reduced 7, 8 and optimism that local elimination of HCV might be achieved 9, real‐world scale‐up of HCV elimination remains largely hypothetical. Though the high price of DAAs has hindered scale‐up 10, treatment levels have also been hindered by ineffective strategies to adequately target key populations who would benefit most from HCV treatment as prevention 11.

The HIV co‐infected population are more likely to be engaged in medical care than the HCV‐mono‐infected population given increasingly high levels of HIV ART uptake globally 12. Theoretically, this provides an opportunity for broad coverage of HCV treatment in this population, particularly given that DAA treatment regimens are similar in co‐infected and mono‐infected populations with equivalent treatment outcomes 13, 14. However, it is important to note that despite increases in ART coverage, coverage in the key at‐risk populations still lags behind in some contexts possibly due to lower levels of engagement in medical care and discriminatory prescribing practices 15, 16.

Despite the advantages of DAA therapy, engaging large numbers of HIV/HCV co‐infected people in HCV DAA treatment will likely require tailored strategies for each of the three key groups at risk of co‐infection: PWID, GBM and prisoners. A number of key barriers will need to be addressed to achieve a comprehensive increase in HCV treatment coverage in these three key populations. Insights from implementation science suggest that the successful and widespread implementation of any innovation is complex, even one that is supported by a high level of evidence 17, 18. This is partially due to barriers at the level of the individual health professional, such as lack of knowledge or skill or negative attitudes, but also involves structural barriers, organizational barriers, peer group barriers (differences between the local standard of care and the desired practice) and professional‐patient interactions 19. In the case of HCV, stigmatization and criminalization of the major patient groups and frequent incarceration of PWID add further to the complexity 20, 21.

There is currently no published real‐world data on the effectiveness of DAA treatment for elimination of HCV infection in HIV‐infected populations at a population level. However, a number of relevant studies and initiatives are emerging. This paper aims to describe and report cascade of care data for emerging HCV elimination initiatives and studies that are currently being trialled in HIV/HCV co‐infected populations in the context of implementation science theory.

## 2 Methods

**Figure 1:**
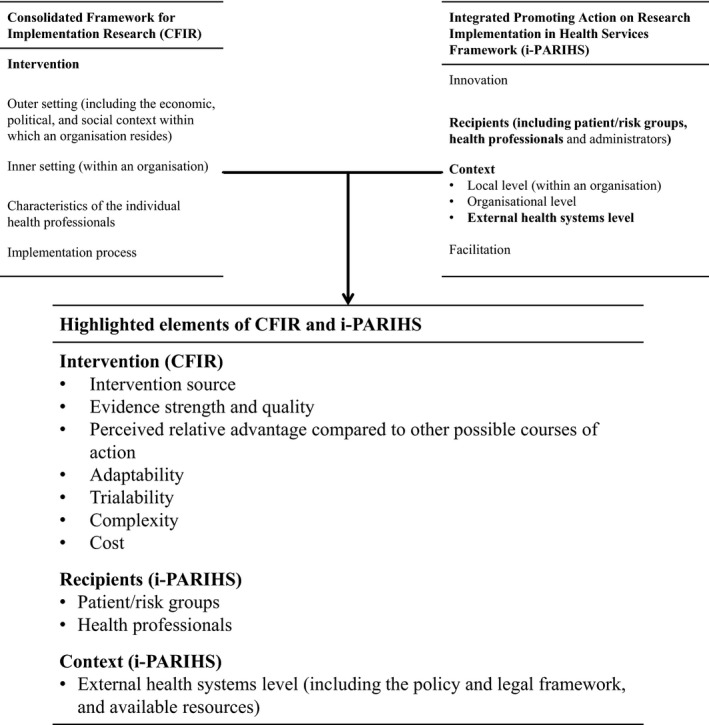
Overview of the CFIR and i‐PARIHS implementation science frameworks with relevant constructs highlighted. While all constructs in the two implementation science frameworks are potentially relevant to HCV elimination interventions, the highlighted constructs are particularly relevant for describing and analysing progress in HCV elimination in HIV‐infected populations, both in the initiatives and studies identified and in the global context.

Seven HCV elimination initiatives and studies in HIV co‐infected populations that are currently underway were identified in six countries. Eligibility criteria for inclusion were either (a) monitoring an HCV elimination intervention targeted to HIV‐infected populations; or (b) monitoring the effects of HCV elimination interventions in HIV‐infected populations. Data on context, intervention characteristics, and initiative/study‐level cascade of care were synthesized using conference abstracts, published manuscripts and personal contact with the investigators. Data collection tools were informed by relevant constructs from the consolidated framework for implementation research (CFIR) 17 and the integrated Promoting Action on Research Implementation in Health Services framework (i‐PARIHS; Figure 1) 18. Cascade of care data from initiatives and studies included the number of people with HCV RNA, the proportion of those with HCV RNA who initiated treatment, the proportion of those who initiated treatment that completed treatment, and the SVR rate. Where possible the SVR rate was defined as the proportion of those who were at least 12 weeks past their expected treatment completion date, who had attained SVR. Data on diagnosis rates were not generally available at the initiative/study level.

For each country in which initiatives and studies were included, model‐based estimates of the numbers of people living with HIV and the proportion diagnosed with HIV were sourced from peer‐reviewed journal manuscripts 22, 23, surveillance reports 24, 25, 26, and conference presentations 27. Estimates of HCV antibody prevalence among HIV‐infected populations were obtained from cohort studies of people living with HIV 26, 28, 29, 30, clinical databases of HIV patients 27, and country‐level HIV/HCV co‐infection management guidelines 31. The number of people affected by HCV/HIV co‐infection was calculated by applying the HCV antibody prevalence estimates to the estimates of the numbers of people living with HIV. An estimate of the proportion of those with HIV/HCV co‐infection who were diagnosed for both HIV and HCV was only available for one country, and it was sourced from a conference presentation 27.

All contributing studies had received ethical approval from local ethics review boards in their countries.

## 3 Results ‐ progress toward HCV elimination in HIV‐infected populations

### 3.1 Emerging research

Seven HCV elimination initiatives and/or studies in HIV‐infected populations were identified, mainly in high‐income countries (Australia (n=2), Canada, France, Georgia, the Netherlands and Switzerland). The broader context in which an intervention takes place is highlighted by the i‐PARIHS framework (Figure 1). The six countries in which elimination initiatives or studies were identified all have harm reduction programmes for PWID, and protection against discrimination for LGBT populations. 32, 33. However, the coverage of harm reduction programmes varies substantially between countries. According to a recent systematic review, needle and syringe programme coverage was high in Australia and the Netherlands (>200 needles/syringes distributed per PWID per year), moderate in Canada, France and Switzerland (100 to 200 needles/syringes distributed per PWID per year) and low in Georgia (0 to 50 needles/syringes distributed per PWID per year). Opioid substitution therapy coverage was high in Australia, France, the Netherlands and Switzerland (>40 recipients per 100 PWID), moderate in Canada (20 to 40 recipients per 100 PWID), and low in Georgia (0 to 20 recipients per 100 PWID) 32. HCV elimination strategies operating at the regional or country level were in place in Australia, France, Georgia and the Netherlands (Table 1).

**Table 1:** Country‐level context of the identified HCV elimination initiatives and studies in HIV‐infected populations

	Australia	Canada	France	Georgia	Switzerland	The Netherlands
Main population groups affected by HIV/HCV co‐infection						
PWID	N	Y	Y	Y	Y	Y^a^
GBM	Y	Y	Y	Y	Y	Y
Prisoners	N	Y	Y	Y	Y	N
Other	N	Y^b^	N	Y	N	N
National/regional HCV elimination strategy	Y	N	Y	Y	N	Y
Availability of subsidized DAAs						
Unrestricted in HIV‐coinfected population (year)	Y (2016)	Y (2014/2017)^c^	Y (2017)	Y (2015)	Y (2017)	Y (2015)
Unrestricted for all chronic HCV patients (year)^d^	Y (2016)	N^e^	Y (2017)	Y (2015)	N	Y (2015)
Prescriber types						
Specialists	Y	Y	Y	Y	Y	Y
Primary care	Y	Y/N^f^	N	N^g^	N	N
Legal constraints for key populations						
Harm reduction programmes for drug use	Y	Y	Y	Y	Y	Y
Protection against discrimination for LGBT populations	Y	Y	Y	Y	Y	Y

a In the Netherlands, HCV/HIV coinfection is mainly observed in former PWID and GBM. Recent transmission has been observed only among GBM. Similar to the situation in other high‐income countries, HCV transmission among GBM is thought to be driven partially by sexual transmission and partially by injecting and non‐injecting drug use to enhance the sexual experience.

b Indigenous populations (mainly through injecting drug use).

c Simeprevir and sofosbuvir were unrestricted in Quebec for HCV mono‐infected patients since 2014. Although HIV infection was a listed restriction, co‐infected patients were usually granted access on a case by case basis through the “patient d'exception” process; Ledipasvir and ombitasvir/paritaprevir/ritonavir; dasabuvir were unrestricted in Quebec from 2016 and velpatasvir from 2017; and the majority of other Canadian provinces since March 2017 34.

d Generally excluding those who are incarcerated.

e Not currently available but this is expected to change in 2018/9 in the majority of Canadian provinces.

f Primary care practitioners can prescribe HCV treatment in some provinces but not others 34.

g A pilot programme is currently underway to evaluate HCV treatment in primary care, which is expected to lead to all primary care providers being allowed to prescribe HCV treatment.

In addition to the broad context, another key aspect is the characteristics of the patient or target group of the intervention (Figure 1). The main population groups affected by HIV/HCV co‐infection in the countries in which elimination initiatives were identified included PWID, GBM and incarcerated populations. However, the majority of the elimination efforts were targeted elimination efforts in GBM. The two Australian initiatives (CEASE and Co‐EC) operate primarily in cities where GBM account for approximately 85% of HIV/HCV co‐infection cases; in Amsterdam, ongoing transmission of HCV infection among HIV‐infected populations is only among GBM; and in Switzerland, the Swiss HCVree Trial is a clinic trial with MSM as an eligibility criterion. Notably, the HCVree study is nested in the Swiss HIV Cohort Study which includes PWID, GBM and other HIV‐infected participants, and because treatment data are not yet available for HCVree, the cascade of care data presented here are from the Swiss HIV Cohort Study. The nationwide HCV elimination programme in Georgia provides HCV treatment at harm reduction sites for PWID, HIV centres, and prisons. In Canada and France, there are no specific programmes targeting HIV‐infected populations but the effects of general HCV elimination programmes are being evaluated in the HIV‐infected community. France's national strategy for HCV elimination includes targeted programmes for PWID. Broad availability of DAAs is the only nation‐wide elimination initiative in Canada but there are other HCV elimination initiatives in Canada that are targeted to specific sub‐populations and geographic areas. The cohort studies and databases being used to evaluate the effects of HCV elimination programmes in Canada, France and Georgia include diverse HIV‐infected populations including active PWID and GBM. (Table 2).

**Table 2:** Intervention characteristics of seven key studies targeting HCV elimination in HIV/HCV co‐infected populations

Name (location)	Scope of intervention	Intervention components	Who covers the cost of the intervention	Evaluation method in HIV‐infected populations
(Canada)	Nation‐wide	Broad access to DAAs; additional clinic‐level, province‐level, and community‐level interventions	Government/health insurance^a^	Canadian co‐infection cohort 40
co‐EC (Melbourne, Australia)	Three high HIV caseload primary healthcare clinics, the largest metropolitan sexual health centre, and the two largest hospitals for care of people living with HIV, accounting for over 75% of people living with HIV in Victoria	Broad access to DAAs and broadened prescriber base; nurse supported programmes to identify patients for HCV testing, and support for GPs to increase testing and treatment; posters displayed in participating clinics promoting HCV testing to patients; nurse‐led model of care in primary healthcare to increase access to DAAs; training programmes for nurses and physicians	DAA therapy is government subsidized; practice nurses are funded by industry through investigator initiated research	An integrated HCV/HIV clinical and behavioural surveillance system monitors the impact of the programme at the local and statewide level
CEASE (predominantly Sydney, Australia)	Nation‐wide observational study of HCV viraemia among HIV‐infected population, with an implementation project predominantly operating in Sydney	Broad access to DAAs and broadened prescriber base; HCV Education for HIV prescribers; recurrent viraemia: monitoring of a cohort of high‐risk inner city patients for reinfection	DAA therapy is government subsidized; other intervention components funded by industry through investigator initiated research	Data assessed at three cross‐sectional visits; at enrolment (2014 to 2016), follow up 1 (2017 to 2018) and follow up 2 (2019 to 2020); data include HCV viraemic prevalence through DBS, behavioural risk and fibrosis assessment
MC FREE (Amsterdam MSM HCV Free, Amsterdam, the Netherlands)	City‐wide	Broad access to DAAs; home‐based HCV RNA dried blood spot testing service (subscription‐based); online tools including information and personal advice on testing and risk reduction strategies, and test results delivered online; motivational interviewing and intensification of partner notification; online and offline media campaigns aimed at increasing HCV awareness; interventions aimed at professionals; behavioural interventions by trained HIV nurses	DAA Treatment is government subsidized; home‐based testing and online/ offline strategies are supported by industry through investigator initiated research	Through the National HIV Monitoring Foundation and the MOSAIC study, the different interventions will be evaluated according to predefined criteria/ deliverables
National Plan for HCV Elimination (France)	Nation‐wide, community‐based intervention	Broad access to DAAs; community‐based test and treat model involving implementation of rapid diagnostics at the community level; educators and social workers who link PWID / marginalized people to healthcare centres, through a case management (*parcours*) programme	Government/health insurance	Several cohorts of HIV‐infected patients and HIV/HCV co‐infected patients in addition to national surveillance systems
National HCV elimination programme (Georgia)	Nation‐wide multi‐component programme	Broad access to treatment and increased access to treatment through primary care, harm reduction sites, HIV centres and prisons^b^; advocacy, awareness and education; harm reduction among PWID, blood safety and infection control in traditional and non‐traditional healthcare settings; national HCV screening Laboratory diagnostics capacity building; surveillance	Gilead and the CDC among others	Georgian National AIDS health information system (AIDS HIS, a secure web‐based system connecting all HIV care providers countrywide)
The Swiss HCVree Trial (Switzerland)	Research study‐based elimination effort among HIV/HCV co‐infected MSM, operating nationwide	Cohort study based test and treat model; delivery of Elbasvir/Grazoprevir treatment to patients infected with genotypes 1 and 4 through clinical trial (Swiss HCVree Trial); behavioural intervention delivered to patients reporting inconsistent condom use with occasional partners; the remaining patients received standard of care and written and oral information on prevention of HCV reinfection.	Investigator initiated trial nested in the Swiss HIV Cohort Study (SHCS); SHCS mainly funded by the Swiss National Science Foundation, HCVree mainly funded by industry	The Swiss HCVree Trial: all study participants tested for HCV RNA at beginning and end of the HCVree trial, change in risk behaviour is evaluated, see ClinicalTrials.gov NCT02785666; effects on HCV incidence within HIV‐infected populations evaluated using the Swiss HIV Cohort Study

a Although all Canadian citizens and permanent residents have insurance coverage for in‐hospital and physician services, medication coverage varies across the 10 provinces and 3 territories, with a mix of both public and private sources of insurance depending on individual characteristics. For example, people on social assistance receive public coverage for medications with no or minimal co‐payments and Indigenous people receive medication coverage from the First Nations and Inuit Health Branch (FNIHB).

b Provision of HCV treatment through primary care and harm reduction sites is currently being piloted and will be implemented nationwide in the future.

One of the characteristics highlighted in the CFIR is the complexity of the intervention (Figure 1). HCV elimination in HIV‐infected populations is complex. All of the initiatives and studies include broad access to DAAs and most include additional components, including screening and testing components, treatment access components, training of health professionals, media campaigns and risk reduction components. Screening and testing interventions include nurse supported programmes to identify patients for HCV antibody testing, community‐based rapid diagnostics and home‐based dried blood spot testing, and study or community‐based reinfection monitoring. Treatment access interventions include a case management programme for PWID and marginalized people, broadened prescriber base, treatment provision at harm reduction sites, treatment provision in prison, nurse‐led primary healthcare based models of care, and community and cohort‐study based test and treat models. Risk reduction components include harm reduction, healthcare‐based infection control, behavioural interventions and personalized online tools for those at risk of reinfection. Of the five broad groups of intervention (screening/testing, treatment access, training of health professionals, media campaigns and risk reduction interventions), MC Free and Georgia's nationwide HCV elimination programme involve components from all five groups, Co‐EC Australia involves components from four groups, CEASE Australia involves components from three groups, the National Plan for HCV Elimination and the Swiss HCVree trial include components from two groups. Although there are no targeted national elimination interventions in Canada, regional interventions such as the Targeted Disease *Elimination™* programme in British Columbia 35 and a pilot project in Big River First Nation, Saskatchewan are evaluating eliminating HCV at the community level. Some of the studies and initiatives include multiple components within a group (Table 2).

The ability to trial the intervention at a small‐scale is also highlighted in CFIR (Figure 1). In the seven initiatives and studies identified, cohort studies, health information systems and surveillance systems are being used to evaluate HCV elimination interventions in people living with HIV (Table 2). None of the evaluation methods include *traditional* control groups. However the CCC (Canada) is using quasi‐experimental designs to (i) evaluate treatment uptake through natural variations in DAA reimbursement policies across Canada and (ii) pilot elimination interventions at the clinic level, comparing intervention sites with matched control sites within the CCC. Similarly, several of the other studies and initiatives are using nationwide databases and/or cohorts to compare regions/sites with interventions to other regions.

The cost and available resources for implementing an intervention are highlighted in CFIR and i‐PAHRIS (Figure 1). With the exception of the Swiss HCVree Trial, where drugs are provided by industry, governments and health insurance provide funding for subsidized DAAs in all of the other HCV elimination initiatives and studies. However, with the exception of the government‐funded National Plan for HCV elimination in France and the nationwide HCV elimination programme in Georgia which is partially industry‐funded and partially funded by the US CDC among others, additional components of the elimination interventions identified were mainly funded by industry through investigator‐initiated research. These components include treatment access interventions, screening/testing, education initiatives for health professionals, media campaigns and risk reduction interventions.

### 3.2 Country‐level burden of HIV/HCV co‐infection and diagnosis

Model‐based estimates of the number of people living with HIV were available for all six countries in which the identified HCV elimination initiatives and studies were based. These ranged from 9600 in Georgia to 149,900 in France. The percent of HIV‐infected participants with HCV antibodies ranged from 12% in the Netherlands to 40% in Georgia. The number of people living with HIV and HCV antibodies ranged from 2600 in Switzerland to 36,400 in France. The percent of HIV‐infected individuals who were HIV diagnosed ranged from 42% in Georgia to 89% in Australia (Table 3). An estimate of the percent of co‐infected individuals who were diagnosed for both HIV and HCV was reported in a conference presentation for Georgia (33%) 27, but was not available for any of the other countries. According to systematic reviews and modelling studies, the estimated proportion of people living with HCV (including those with HCV monoinfection) who were diagnosed prior to DAA availability was 37% (Switzerland), 57% (France), 61% (the Netherlands), 70% (Canada), 75% (Australia) 36, 37, 38, 39. However, in some countries these diagnosis rates may be quite different in the HIV‐infected population.

**Table 3:** Country‐level burden of HIV/HCV co‐infection and diagnosis

	Australia	Canada	France	Georgia	Switzerland	The Netherlands
Estimated number of people living with HIV	26,400 24	65,000 25	149,900 22	9600 27	15,200 23	22,900 26, 41
Estimated % with HCV antibodies	13 28	20 to 30 31	24 29	40	17 30	12 26
Estimated number with HCV antibodies	3500	16,300	36,400	3900	2600	2700
Estimated % of those living with HIV who are HIV diagnosed	89 24, 28	80 25	81 22	42 27	81 23	89 26, 41

All numbers are rounded to the nearest 100 and percentages are rounded to the nearest percent. Estimated numbers with HCV antibodies are calculated by applying the estimated % with HCV antibodies to the estimated number of people living with HIV. Percent HIV diagnosed is among all HIV‐infected people.

### 3.3 Initiative‐level partial cascade of care

**Figure 2:**
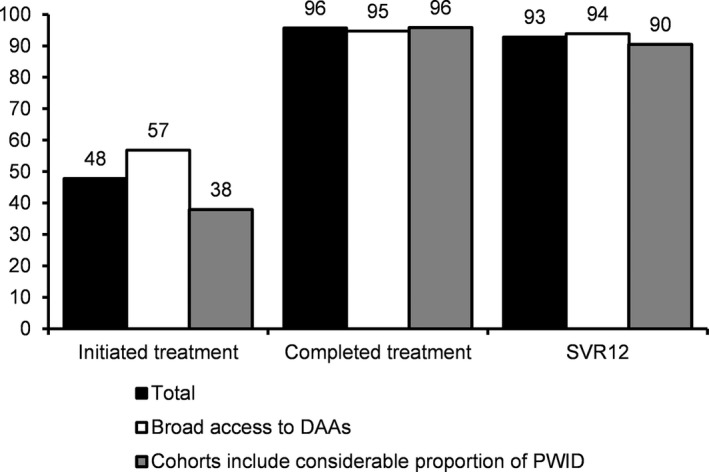
Partial cascade of care data from seven initiatives and studies implementing and evaluating HCV elimination interventions in HIV‐infected populations. Percent of HCV RNA positive participants in care or enrolled in cohort studies initiating treatment, percent of those initiating treatment who have completed treatment, and percent of those who can be assessed for SVR12 who attained SVR12.

Overall, HCV RNA was detected in 4894 HIV‐infected participants across the seven studies and initiatives (range within studies: 297 to 994). Of these 2338 initiated HCV treatment (48%; range: 21% to 85%). Among studies with treatment completion data, 1061 of 1109 initiating treatment (96%, range: 94% to 99%), completed treatment. Of those who were treated with DAAs and could be assessed for SVR12, 1631 of 1757 attained SVR12 (93%, range: 86% to 98%). Of the seven studies and initiatives, four reported cascade of care data from a period where DAAs were broadly available (CEASE and Co‐EC ‐ Australia, ATHENA ‐ The Netherlands and AIDS HIS‐Georgia), and three included data from prior to DAAs becoming broadly available (CCCS ‐ Canada, SHCS ‐ Switzerland, and HEPAVIH‐ France). Four initiatives/studies included a considerable proportion of PWID in addition to GBM and other patient groups (CCCS, AIDS HIS, HEPAVIH and SHCS), and three were composed mainly of GBM and former PWID (CEASE, Co‐EC and ATHENA). In studies and initiatives with cascade of care data from a period where DAAs were broadly available, 1398 of 2460 (57%, range: 36% to 74%) initiated HCV treatment, 160 of 169 (95%: data from one study only) completed HCV treatment, and 1042 of 1110 (94%, range: 88% to 98%) attained SVR12. In studies and initiatives that include a considerable proportion of current PWID, 1271 of 3349 (38%, range: 21% to 85%) initiated treatment, 901 of 940 (96%: range: 94% to 99%), completed HCV treatment, and 885 of 978 (90%, range: 86% to 96%) attained SVR12; however, two of these studies and initiatives include data from prior to DAAs becoming broadly available (Table 4 and Figure 2). These data include diagnosed patients, who are in care (France, the Netherlands, Sydney), were in care previously (Melbourne, Georgia) and/or are enrolled in a cohort study (Canada, France, the Netherlands and Switzerland).

**Table 4:** Partial cascade of care in the HCV elimination interventions/studies identified in HIV‐infected populations

	CEASE Australia	Co‐EC Australia	CCC Canada	HEPAVIH France	AIDS HIS Georgia^a^	SHCS^b^ Switzerland	ATHENA^c^ The Netherlands
Number HCV RNA positive	297^d^	305^e^	994^f^	564	915	876	943^g^
Number initiated HCV treatment (%)^h^	196 (66)	169 (55)	278 (28)	482 (85)^i^	331 (36)	180 (21)	702 (74)
Number completed HCV treatment (%)^j^	‐	160 (95)	271 (97)^k^	451 (94)	‐	179 (99)	‐
Number attained SVR12 (%)	15 (88)^l^	133 (89)^m^	240 (86)^n^	176 (93)^o^	296 (89)	173 (96)	598 (98)^p^

CEASE, Control and Elimination within AuStralia of HEpatitis C from people living with HIV; co‐EC Study, Eliminating HCV/HIV co‐infection; CCC, Canadian Co‐infection Cohort; HEPAVIH, the French national prospective cohort of patients co‐infected with HIV and HCV (ANRS CO13 HEPAVIH); AIDS HIS, AIDS health infection system; SHCS, Swiss HIV Cohort Study; ATHENA, AIDS Therapy Evaluation in the Netherlands (ATHENA) cohort.

a Data from the Georgian National AIDS health information system (a secure web‐based system connecting all HIV care providers countrywide), June 2015 to August 2016. DAAs were broadly available.

b Cascade of care in the Swiss HIV Cohort Study because cascade of care data are not yet available for the Swiss HCVree Trial. Data are from 2014 to 2015. Second‐generation DAAs were available but restricted by liver disease stage 42.

c Cascade of care from the ATHENA cohort of people diagnosed with HIV in the Netherlands from a period where DAAs were broadly available but prior to MC Free, Amsterdam 45. A cascade of care at the two largest HIV clinics in Amsterdam (a subset of the ATHENA cohort) is available for the same period 46.

d 2014 to 2016 47.

e These are patients who have been tested for HCV in the past but may not currently be in HCV care. Among those who have been tested for HCV within the study period (n=194), the treatment uptake is 87%.

f As of 21 November 2013 (Health Canada's approval of second‐generation DAAs), cohort participants who were eligible to initiate DAAs.

g Includes those who have tested HCV RNA positive, were treated with DAAs, have never been treated, or were treated with interferon‐based therapies and failed treatment but have not yet been retreated 45.

h Of those HCV RNA positive.

i Includes initiations with interferon‐based therapies during a period when DAAs were available in France but restricted 48.

j Of those initiating HCV treatment.

k Data collected up to December 2016.

l Up to end 2015 (17 had initiated treatment).

m Of those who are 12 weeks past the date of planned treatment completion. 136 of these were tested for HCV RNA at 12 weeks after treatment, and of those the SVR rate was 98%. It is likely that more participants will be tested for HCV RNA at their next HIV clinic visit.

n Includes DAA regimens including interferon, data collected up to July 2017 43.

o Of those initiating DAA therapy prior to February (24 week therapy)/May 2015 (12 week therapy) 49.

p Of those who were assessed for SVR.

## 4 Discussion

Early results were synthesized from seven HCV elimination initiatives and studies in HIV‐infected populations. These results demonstrate increased linkage to HCV care, successful retention in care, and high cure rates among those diagnosed with HCV/HIV co‐infection. However, these are early data and the majority of initiatives and studies identified were in high‐income countries with relatively low levels of criminalization of risk behaviours and discrimination and stigma of PWID and GBM. Furthermore, the majority of initiatives and studies identified were either primarily treating GBM and/or former PWID or include data from prior to broad DAA availability. None of the studies operate in incarcerated populations and treatment in this context may be more challenging. More data will be required to evaluate the effects of treatment scale‐up on HCV prevalence and incidence, and confirm that these high rates of linkage and retention in care can be replicated in PWID, incarcerated populations, and in countries with greater levels of criminalization of risk behaviours and discrimination and stigma of the groups at risk. Different strategies may be required for linkage and retention in care in different populations.

Overall, approximately 50% of HCV diagnosed individuals in the seven initiatives and studies were linked to HCV treatment. This represents a substantially higher treatment uptake than prior to DAA therapies becoming available. In the Swiss HIV cohorts, treatment uptake increased fourfold after the introduction of second‐generation DAAs 42. In Georgia, approximately 100 HCV/HIV co‐infected people were treated per year in the three and a half years prior to DAA availability, compared to approximately 265 treatments/year in the first 15 months of the nationwide HCV elimination programme 27. In the CCC (Canada), treatment uptake increased from 8 per 100 person years to 28 per 100 person years after the introduction of DAA therapies. 43. This is consistent with increases in treatment uptake following the introduction of DAA therapies in predominantly HCV‐monoinfected populations 44. However, it is not clear that these increases in treatment rates will be sustained. In the Netherlands, treatment numbers increased substantially from November 2015 when DAA therapy became available until July 2016 (on average, >150 treatments/quarter) but then returned to pre‐DAA levels (<50 treatments/quarter) 45. Moreover, although substantial increases in treatment uptake were observed after the introduction of DAA therapy, treatment uptake varied substantially between studies (21% to 91%). This is partially due to variations in when broad access to DAA therapy was attained: in Canada, France, and Switzerland, although DAA therapies were available to selected subgroups earlier, broad access to DAA therapies was only attained in 2017 and cascade of care data was either not yet available or included data from before broad access was attained. Treatment rates may have increased since DAAs became broadly available.

However, even in initiatives and studies that attained broad access to DAA therapies earlier, treatment uptake ranged from 36% in Georgia to 75% in the Netherlands indicating that there are significant barriers to care other than simply access to DAA therapy, including provider‐level, patient‐level and structural barriers (19, 20, 21, Table 5). Overall, more than half of diagnosed HIV/HCV co‐infected individuals had not received treatment. While the data included only reflect a short timeframe after the introduction of DAAs, this suggests that substantial effort is still required to achieve HCV elimination in HIV‐infected populations. In order to overcome the potential barriers to HCV elimination, it is likely that complex interventions will be required. These are likely to include interventions related to linkage and retention in care, diagnosis and screening, training of health professionals, risk reduction and identification and treatment of reinfection cases. Notably, currently the majority of intervention components in the identified studies and initiatives other than DAA therapy are funded by industry through investigator‐initiated research. In order to attain HCV elimination globally, sustainable financing structures will be required to monitor the effectiveness of HCV elimination efforts and for widespread implementation of interventions that are proven to be effective.

**Table 5:** Barriers and enablers of HCV elimination in HIV‐infected populations: list structure based on the CFIR and i‐PARIHS frameworks

Intervention	
Strength of evidence	High level of evidence that DAAs are efficacious and tolerable in HIV‐coinfected individuals 13, model‐based evidence of the effectiveness of treatment as prevention 7, 8.
Complexity	Complex intervention targeting populations rather than individuals, and involving case finding, engagement and retention in care, follow‐up after successful treatment, and potential re‐treatment following re‐infection.
Adaptability	Case finding strategies and models of HCV care are adaptable 50, 51.
Trialability	Small‐scale trials are challenging because of the interlinked nature of the transmission networks. In many contexts HCV elimination will only be testable through before and after studies of population‐wide interventions.
Cost	Initially extremely costly; however, some countries have negotiated better rates or early introduction of generics and prices of DAAs are evolving rapidly 10, 52.
Source	Targets have been set by the WHO 9.
Relative advantage	In settings where resources are limited relative to the cost of DAAs, treatment of those with advanced liver disease has been prioritized over those at risk of transmission 52.
Recipients of the intervention	
Healthcare professionals	Negative attitudes of healthcare providers toward risk groups have been documented in some contexts, with consequences for quality of care 16, 53.Possible low levels of knowledge of HCV among primary care practitioners managing HIV patients 54
Patients	Linkage to healthcare and treatment readiness in key population groups, including possible mistrust of healthcare providers among key risk groups 20
	Transience of key populations, particularly PWID, including frequent short‐term incarceration 20, 55 Priority of HCV care in the context of comorbidities and socio‐economic disadvantage 20
Relationships between healthcare professionals and patients	Communication difficulties between patients and specialists 20
Context	
Criminalization, discrimination and stigma of key populations	Criminalization of same‐sex sexual acts in 75 countries 33. High or medium levels of discrimination against gay and bisexual men limit access to HIV‐care in low and middle‐income countries in all global regions 56, limiting access to care for HCV in HIV health services. Criminalization of drugs is widespread, with decriminalization of some aspects of drug use only in relatively few countries, approximately 25 to 30 worldwide. There is some evidence that criminalization of drugs and stigma against PWID in healthcare settings negatively impact on HIV prevention and healthcare 53, 57, 58, and may impact negatively on HCV treatment among HIV‐infected populations.
Incarceration of key populations	Incarceration of PWID and less commonly of GBM poses challenges not only for HIV and HCV‐prevention, but also delivery of HCV treatment 59. While prisons may potentially provide opportunities for HCV treatment, there are also potential challenges to providing continuity of care in the context of prison transfers and short sentences, lack of family support, high levels of stress, unpleasant healthcare context and stigmatization by other inmates and custodial staff 55. In addition, high rates of reinfection have been observed in the prison setting, highlighting the importance of offering other harm reduction interventions such as OST alongside HCV treatment 60, 61.
Health systems and regulatory frameworks	Prescriber‐type restrictions for HCV treatment 62, and lack of access to transient elastography 54 may impact on access to care 20
Resourcing	Resourcing limitations within countries are a function of drug prices (which vary substantially between countries), epidemic size and available resources. In addition to funding of drugs, screening, health‐systems and risk reduction intervention costs also need to be considered.
Formal endorsement at a country or regional level	While HCV elimination is recommended by the WHO, formal endorsement at a country or regional level varies between countries.

All treatment linkage data reflect HIV and HCV diagnosed populations. Population‐wide proportions of those diagnosed with HIV/HCV co‐infection who have received treatment are likely to be lower than those presented here. HCV diagnosis rates among HIV‐infected populations are not currently well understood and additional research is required to estimate these. Two of the initiatives and studies identified included interventions to increase HCV diagnosis in HIV diagnosed populations using community‐based rapid diagnostics and home‐based dried blood spot testing. Dried blood spot testing administered by professionals has previously been found to increase HCV testing uptake in populations at high risk of HCV monoinfection 63. In Georgia, where the rate of HIV diagnosis is relatively low (approximately 40%), efforts are being made to increase diagnosis through integration of HIV, HCV and TB testing services 64.

It is likely that appropriate strategies to improve linkage to care and maintenance in care will differ by population group regardless of whether they are HIV/HCV co‐infected or HCV monoinfected. For PWID, prior to the introduction of DAA therapy, a meta‐analysis of determinants of HCV treatment completion and efficacy in drug users found that addiction treatment and support services during HCV therapy predicted treatment completion, and the involvement of a multidisciplinary team predicted SVR 51. A subsequent meta‐analysis that included studies of drug‐using and other populations found that coordinated mental health, substance use and HCV treatment services had a modest effect on treatment completion and SVR but not on treatment uptake. The level of evidence was rated as very low on the GRADE scale although that is partially due to the difficulties of conducting blinded randomized controlled trials of these interventions. The same meta‐analysis failed to find any effect of directly observed therapy on SVR 50. Since the introduction of DAAs, conference abstracts describing a range of models of care for PWID including directly observed therapy 65, 66, addiction treatment and support services during HCV therapy 67, 68, support groups 65, integration of HCV treatment clinic and harm reduction services 69, community‐based clinic conducting outreach at rehab clinics 70 have all reported high levels of SVR in PWID with HCV mono‐infection.

Four of the HCV elimination initiatives identified in this study are implementing models of care targeting GBM. These involve integrating HCV treatment with HIV care, and targeted risk reduction strategies. Prevention of reinfection was highlighted as an important component of HCV elimination efforts in HIV‐infected GBM in a mathematical model based on data from the Swiss Cohort study. In light of empirical evidence of increases in HCV‐related risk behaviours in GBM being enrolled in the Swiss Cohort Study, the model suggested that if the trend toward increasing risk behaviours persist, high rates of reinfection will mean that even very high treatment rates will not result in reductions in HCV incidence unless treatment is combined with behavioural interventions to reduce risk behaviours after treatment 71. The HCVree study includes an RCT trialling a behavioural intervention to prevent reinfection. Results are not yet available.

Prisoners are another complex population likely to require targeted approaches for HCV elimination. More research is required to understand strategies for linkage to care in this context. In addition, indigenous peoples, heterosexuals infected with HIV, and migrants from high prevalence HIV and HCV countries are other groups that may also require targeted approaches for linkage to care and maintenance in care. Further research is also required to understand the impact on HCV elimination strategies in HIV‐infected populations of reinfection following successful HCV treatment 72, 73, transmission of HCV between HIV infected and uninfected populations 74 and migration‐ and travel‐related transmission of HCV between countries 75. Furthermore, randomized controlled trials are needed to evaluate the efficacy of strategies to enhance diagnosis, linkage to care and maintenance in care, and risk reduction where feasible and ethical.

This study has a number of limitations. HCV elimination initiatives and studies in HIV‐infected populations were not identified through a systematic search and the list presented here is not exhaustive. As previously indicated data on treatment linkage were all early data and represent different stages of DAA treatment availability.

Early data from the DAA era suggest that HCV treatment uptake has increased in HIV‐infected populations compared to previous levels, but there is still considerable work to do on the pathway to HCV elimination in this population. This includes efforts to quantify the numbers of undiagnosed infections, and increase diagnosis rates and linkage to care. It is likely that different strategies will be required for different populations including PWID, GBM and prisoners among others. Among those who have been treated with DAAs, treatment completion and treatment success has been consistently high across a variety of settings.

## Competing interests

JSD and MEH report grants from Gilead Sciences, Abbvie and BMS to support investigator initiated research. JD and JD's institution have also received honoraria from Gilead, BMS and MSD. AR and CB report grants from MSD during the conduct of the study to support investigator initiated research. GVM reports grants from Abbvie, Gilead Sciences and BMS to support investigator initiated research. MP's institute received speaker's fees and unrestricted grants that contribute to several research projects from Gilead, Roche, MSD and Abbvie. MvdV's institute received speaker's fees and unrestricted grants that contribute to several research projects from Gilead, Roche, MSD and Abbvie. MBK has received research grants for investigator‐initiated trials from Merck and ViiV Healthcare; consulting fees from ViiV Healthcare, Bristol‐Meyers Squibb, Merck, Gilead and AbbVie. FLA reports grant support from the Gilead Foundation, Merck, NIDA, NIAAA, SAMHSA and HRSA, speakers bureau fees from Gilead Sciences, and advisory board/consultation fees from Gilead, Merck, and BMS. RSD, AP, SS, KL, and NC have no competing interests to declare.

## Authors’ contributions

RSD, JSD, AR, FLA and MEH designed the data collection tool and manuscript concept; JSD, AR, CB, GVM, MP, MvdV, MBK, SS, KL, NC and MEH provided data on their HCV elimination in HIV co‐infected populations initiatives and studies; RSD wrote the first draft of the manuscript; JSD, AR, CB, AEP, GVM, MP, MvdV, MBK, SS, KL, FLA, NC and MEH read and critically reviewed the manuscript draft.

## Funding

Co‐EC is funded by BMS. HCVree is funded by MSD. The Swiss HIV Cohort study is funded by the Swiss National Science Foundation (grant no. 148522), and by the SHCS Research Foundation. CEASE is funded by Gilead Sciences and BMS. MC Free is funded by Abbvie, Gilead Sciences, Johnson & Johnson, Merck Sharp & Dohme, and Roche. The Canadian Co‐infection Cohort Study is funded by the Canadian Institutes of Health Research (CIHR; FDN‐143270), the CIHR Canadian HIV Trials Network (CTN‐222) and the Fonds de recherche Québec‐ Santé, Réseau SIDA/maladies infectieuses (FRQ‐S). Georgian AIDSHIS is co‐funded by the Georgian national HIV/AIDS Management Program and the Global Fund to Fight AIDS, Tuberculosis and Malaria. ANRS CO13 HEPAVIH is funded by the French National Agency for Research on AIDS and Viral Hepatitis (ANRS) and also receives funding from Roche, Schering‐Plough, GSK, BMS, and Merck‐Serono. RSD is supported by an Early Career Fellowship from the Australian National Health and Medical Research Council. JD is supported by a Clinical Fellowship from the Australian National Health and Medical Research Council. MBK is supported by a Chercheur National career award from the FRQ‐S. MH is supported by a Principal Research Fellowship from the Australian National Health and Medical Research Council. The Burnet Institute is supported by the Victorian Operational Infrastructure Support Program.
